# Use of a recombinase polymerase amplification commercial kit for rapid visual detection of *Pasteurella multocida*

**DOI:** 10.1186/s12917-019-1889-6

**Published:** 2019-05-17

**Authors:** Guimin Zhao, Hongbin He, Hongmei Wang

**Affiliations:** Ruminant Disease Research Center, Key Laboratory of Animal Resistant Biology of Shandong, College of Life Science, Shandong Normal University, No.88 Wenhua East Road, Lixia District, Jinan, 250014 Shandong Province China

**Keywords:** Recombinase polymerase amplification, *Pasteurella multocida*, Lateral flow dipstick

## Abstract

**Background:**

*Pasteurella multocida (P. multocida)* is a bacterium that causes bovine respiratory disease (BRD) and haemorrhagic septicaemia (HS) in cattle, buffaloes and bison. Rapid point-of-care diagnosis or regular testing of Pasteurellosis, therefore, could contribute greatly to early detection, and screening infected animal is important. Up to now, there are no published reports on the use of recombinase polymerase amplification (RPA) combined with a lateral flow dipstick (LFD) for *P. multocida* detection.

**Results:**

This study proposes a promising isothermal detection method for *P. multocida* with the potential to be developed as an on-site test for Pasteurellosis. The method includes an RPA combined with LFD*.* First, the analytical sensitivity and specificity of *P. multocida* RPA-LFD were tested. The RPA-LFD, performed at 39 °C, successfully detected *P. multocida* DNA in 30 min, with a detection limit of up to 120 copies per reaction. Then, the practicability of RPA-LFD was analysed using 62 nasal swabs and 33 fresh lungs samples from 17 different dairy farms. Compared to real-time quantitative PCR (qPCR), the RPA-LFD assay yielded a clinical specificity of 95.15%, positive predictive value (PPV) of 95.15% and 0.958 kappa coefficient. Compared with the culture method, it achieved 100% sensitivity, 67.20% specificity and a 0.572 kappa coefficient.

**Conclusions:**

These results combined with the simple conditions required for the performance of the RPA-LFD assay, have demonstrated the effectiveness and practicability of the method for development into a regular on-site protocol for the diagnosis of Pasteurellosis.

## Background

*Pasteurella multocida* (*P. multocida*) is a pathogenic gram-negative bacterium that plays a role in multihost diseases [1]. *P. multocida* has been identified in shipping fever of weaned calves and in enzootic neonatal calf pneumonia [2]. It also cause heamorrhagic septicaemia (HS), a disease normally found in some areas of Asia, Africa, the Middle East and southern Europe in cattle, buffaloes and bison [3]. Epidemic outbreaks of these diseases can cause serious economic losses in related industries. Development of a quick, exact field diagnostic test for the presence of the pathogen is thus very important for the prevention and control of Pasteurellosis.

At present, nucleic acid amplification technologies (NAAT) are often used in rapid diagnostic tests of infectious diseases. However, most nucleic acid detection technologies with usable sensitivity and accuracy, such as PCR and real-time quantitative PCR (qPCR), require fully equipped stationary laboratories and complex thermal cycling instruments. In addition, most NAAT assays, including a number of isothermal amplification techniques, need power-dependent instruments to provide thermal energy. The reaction time is about 2 h, which is quite time-consuming [4].

Since the first report in 2006, isothermal recombinase polymerase amplification (RPA) nucleic acid amplification technology has been acknowledged for its advantages including rapidity and simplicity [5, 6]. The combination of the RPA assay and a lateral flow dipstick (LFD) is specially suitable for on-site detection of clinical samples. RPA can tolerate temperatures from 30 to 45 °C but does not lose its response efficiency. It has been successfully used for rapid, sensitive and visual detection of bovine rhinotracheitis virus, foot-and-mouth disease virus and *Mycoplasma bovis* in cattle [5, 7, 8]. In this paper, we established an RPA-LFD assay targeting a previously identified conserved gene of *P. multocida*: the *Kmt1* gene [9]. Additionally, we estimated the specificity, sensitivity and clinical application of the RPA-LFD assay in comparison with qPCR and culture methods to assess its practicality and performance as a potential on-site field test.

## Results

### Determination of RPA-LFD conditions

**Fig. 1:**
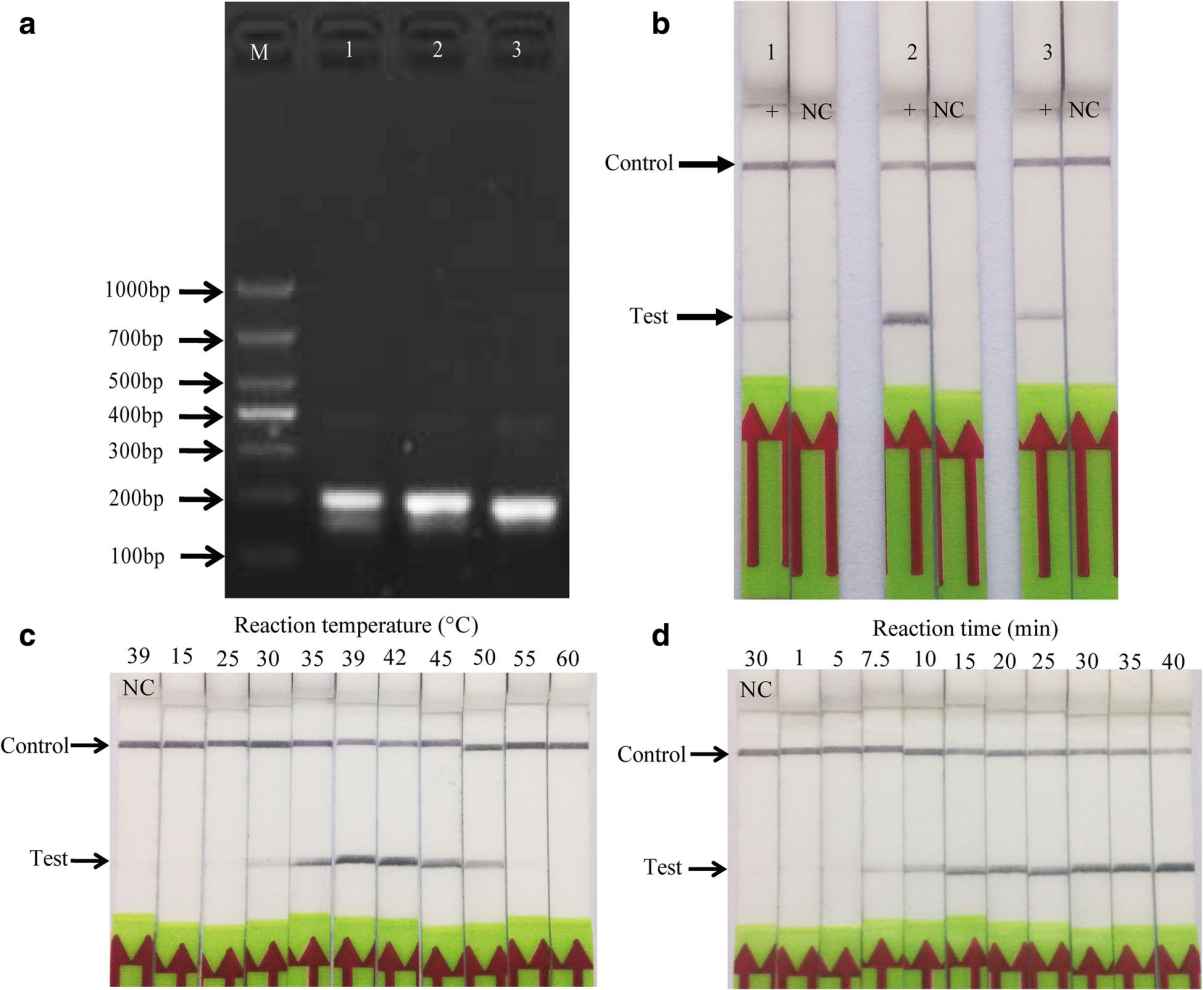
Screening of *P. multocida* RPA-LFD primers and LF-probe. **a** The results of an RPA-nfo reaction for three primer set and LF-probe combination were detected by agarose-gel electrophoresis (lane 1: F1-R, 192 bp; lane 2: F2-R, 189 bp; F3-R, 179 bp; LF-R, 150 bp; M: molecular weight standard DNA marker 1000); **b** ‘+’ showed the results of the RPA-nfo reaction by LFD test, the DNA template was derived from 6 × 10^4^ copies/μl standard DNA, and ‘NC’ was the negative control for the corresponding combination of primers and probe. **c** Optimisation of RPA reaction temperature. The results were positive for reactions performed in temperatures between 35 and 50 °C. **d** Evaluation of amplification time. After a 15-min amplification reaction, the test positive line was clearly visible on the test strip. Lane NC: negative control. Each sample was independently tested three times and one reaction was displayed

First, the analytical specificity of three different combinations of RPA primers and an LF-probe was determined using 6 × 10^4^ copies/μl of standard DNA by agarose-gel electrophoresis (AGE). Two bands were clearly observed by electrophoresis (Fig. 1a). Various primer combinations and targets were evaluated and one primer pair, F2: 5′-TTGCCGCGAAATTGAGTTTTATGCCACTTG-3′ and R: 5′-Biotin-AATAACGTCCAATCAGTTGCGCCGTTGTCA-3′, yielded highest amplification accuracy and was selected during the process, resulting in an amplicon with a size of 189 bp targeting a region on the *Kmt1* gene of *P. multocida* (Fig. 1a). This primer set also produced a faster and darker positive line than the other two groups within 5 min, while no band was noticeable in the negative control (Fig. 1b).

The preferred temperature and time for the RPA reaction were also evaluated. A reaction temperature range of 15–60 °C and a reaction time range of 1–40 min were screened using 6 × 10^4^ copies of plasmid DNA as template. The results showed that the RPA reaction adapted to a broad temperature range of 30–50 °C, and the test band was brightest between 39 and 42 °C (Fig. 1c). Therefore, 39 °C was selected as the optimum reaction temperature in subsequent RPA-LFD testing. Regarding the incubation time, obvious bands were seen at the location of the test area within the range of 15–40 min. However, the band was very weak from 7.5 min to 10 min (Fig. 1d). According to the results, an amplification time of 30 min was selected for all subsequent RPA-LFD assays.

### Sensitivity and specificity of the RPA-LFD assay

**Fig. 2:**
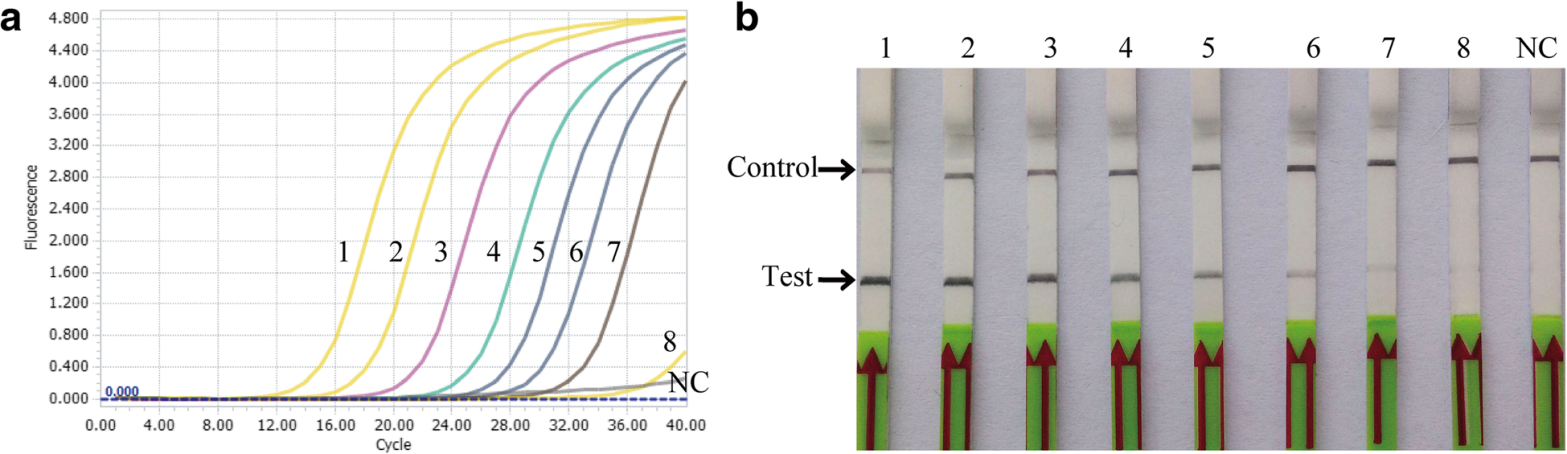
Detection of 10-fold serially diluted *P. multocida* standard DNA by RPA-LFD and qPCR assays. **a** qPCR results. **b** RPA-LFD results. Lane 1 to 8: 10-fold serially diluted *P. multocida* standard DNA from 6 × 10^7^ to 6 copies/μl. Lane NC: negative control. Each sample was independently tested three times and one reaction was displayed

The analytical sensitivity of the *P. multocida* RPA-LFD assay was confirmed using 10-fold serially diluted standard DNA (6 × 10^7^ to 6 copies/μl). The result revealed that the established RPA-LFD assay had a similar molecular sensitivity to that of the qPCR assay with a limit of detection of up to 120 copies per reaction (Fig. 2 and Table 1). Next, the analytical specificity of the RPA-LFD was tested using members of the family Pasteurellaceae and other important bacterial pathogens of cattle which cause similar clinical signs (Table 2). It was determined that no cross-amplification was observed against other pathogens (data not shown). These results indicated that the *P. multocida* RPA-LFD assay was specific for detection of *P. multocida*.

**Table 1:** Assay detection results from *P. multocida* plasmid DNA standards

Number	DNA standards (copies/μl)	Real-time qPCR			RPA-LFD		
		Ct 1	Ct 2	Ct 3	Test 1	Test 2	Test 3
1	60,000,000	13.82	13.95	13.92	+	+	+
2	6000,000	17.15	17.18	17.23	+	+	+
3	600,000	20.12	20.36	20.43	+	+	+
4	60,000	23.92	23.56	23.29	+	+	+
5	6000	26.81	27.07	26.31	+	+	+
6	600	28.19	28.1	28.22	+	+	+
7	60	31.47	31.86	31.09	+	+	+
8	6	40.00	40.00	39.07	–	–	+
9	NC	40.00	40.00	40.00	–	–	–

*Ct* threshold cycle, *NC* negative control

**Table 2:** Bacterial strains used to test RPA-LFD assay specificity

Number	Species	Strains/origin	RPA-LFD
1	*Pasteurella multocida* (A)	BNCC126487^a^	Positive
2	*Pasteurella multocida* (A) JN14	Clinical separation^b^	Positive
3	*Pasteurella multocida* (A) TJ14	Clinical separation^b^	Positive
4	*Pasteurella multocida* (A) JN15	Clinical separation^b^	Positive
5	*Pasteurella multocida* (B)	BNCC126234^a^	Positive
6	*Pasteurella multocida* (B) TJ16	Clinical separation^b^	Positive
7	*Pasteurella multocida* (D)	CVCC392^c^	Positive
8	*Pasteurella multocida* (E)	CVCC393^c^	Positive
9	*Pasteurella multocida* (F)	CVCC394^c^	Positive
10	*Pasteurella avium*	BNCC128728^a^	Negative
11	*Pasteurella gallinarum*	BNCC131586^a^	Negative
12	*Pasteurella trehalosi*	TCCPTA-3668^a^	Negative
13	*Pasteurella stomatis*	ATCC43327^a^	Negative
14	*Pasteurella langaa*	ATCC43328^a^	Negative
15	*Pasteurella dagmatis*	ATCC51570^a^	Negative
16	*Actinobacillus pleuropneumoniae*	BNCC129808^a^	Negative
17	*Mycoplasma bovis* PG45	ATCC25523^a^	Negative
18	*Mycoplasma mycoides* subsp. *mycoides*	BNCC126186^a^	Negative
19	*Mannheimia haemolytica*	BNCC128674^a^	Negative
20	*Trueperella pyogenes*	Clinical separation^b^	Negative
21	*Histophilus somni*	Clinical separation^b^	Negative
22	*Klebsiella pneumoniae*	BNCC194477^a^	Negative
23	*Proteus mirabilis*	BNCC107943^a^	Negative
24	*Streptococcus bovis*	ATCC33317^a^	Negative
25	*Staphylococcus aureus*	ATCC6538P^a^	Negative
26	*Escherichia coli* O157:H7	BNCC186579^a^	Negative
27	*Pseudomonas aeruginosa*	Clinical separation^b^	Negative
28	*Salmonella typhimurium*	BNCC108207^a^	Negative
29	*Enterococcus faecalis*	ATCC29212^a^	Negative
30	*Bacillus cereus*	BNCC103930 ^a^	Negative

^a^These strains were provided from BNCC; ^b^These strains were preserved in our laboratory; ^c^These strains were provided from CVCC

### Performance of RPA-LFD assay on clinical samples

Finally, the practicality and efficiency of the RPA-LFD were compared to those of the qPCR and culture methods. The result showed that RPA-LFD had a similar positive rate compared with that of the qPCR assay [positive rate of 54.74% (52/95) and 52.63% (50/95), respectively (Table 3)]. However, the positive rate of isolated *P. multocida* was lower than that of the RPA-LFD assay in the 95 clinical samples [positive rate of 32.63% (31/95) and 54.74% (52/95), respectively (Table 3)]. The optimized RPA-LFD assay achieved 67.20% specificity, 59.62% positive predictive value (PPV) and a 0.572 kappa coefficient when compared with the bacterial isolation culture method (Table 4). To confirm whether or not the system amplified the correct target, the amplicons of the samples that tested positive in the RPA-LFD assay but negative in the culture or qPCR method were purified and further sequenced. The amplification region of a 189-nucleotide were 100% consistent with that of *P. multocida Kmt1* gene, which proved the accuracy of the RPA-LFD assay.

**Table 3:** Comparison of the *P. multocida* RPA-LFD, real-time qPCR and culture methods on clinical samples

Samples type	Number of samples	RPA-LFD		Real-time qPCR		Culture	
		Positive	Negative	Positive	Negative	Positive	Negative
Nasal swabs	62	29	33	27	35	18	44
Fresh lungs	33	23	10	23	10	13	20
Total	95	52	43	50	45	31	64

**Table 4:** Sensitivities, specificities, kappa values, and positive or negative predictive values of the RPA-LFD and real-time qPCR or culture methods for the detection of *P. multocida*

		Real-time qPCR			Culture		
		Pos	Neg	Total	Pos	Neg	Total
RPA-LFD	Pos	50	2	52	31	21	52
	Neg	0	43	43	0	43	43
	Total	50	45	95	31	64	95
		Sen:100%	Spe:95.15%	K:0.958	Sen:100%	Spe:67.20%	K:0.572
		PPV:96.15%	NPV:100%		PPV:59.62%	NPV:100%	

*Neg* Negative, *Pos* Positive, *Spe* Specificity, *Sen* Sensitivity, *K* Kappa value, *NPV* negative predictive value, *PPV* Positive predictive value

## Discussion

*P. multocida* has been isolated from a number of different species, is potentially zoonotic [10, 11] and has been associated with many different diseases, including BRD. BRD can result in significant economic loss to cattle herds both locally and internationally [12]. *P. multocida* is an extraordinary important pathogen in BRD and has been associated with this disease since the early 1950s [10]. Nevertheless, although an opportunistic role is assumed for this pathogen, many virulence factors and the prevalence of BRD co-infections are recognized [10]. Thus, a robust diagnostic assay capable of rapid, specific and sensitive detection of *P. multocida* in a feedlot or cow-calf operation could play a significant role in reducing the shipping fever of weaned calves and calf pneumonia-related morbidity and mortality, particularly in developing countries.

In this paper we have confirmed the application of RPA-LFD to diagnose Pasteurellosis from clinical samples. An RPA-LFD assay targeting the *Kmt1* gene was examined and demonstrated the ability to detect *P. multocida* DNA with a high degree of specificity and sensitivity in less than 30 min. The sensitivity indicated that the RPA-LFD assay had a similar detection limit to that of the qPCR method (Fig. 2 and Table 1). Furthermore, analytical specificity showed that the assay could detect five distinct capsular serogroups, but detection of common pathogens in infected cattle was negative (Table 2).

To confirm the diagnostic practicality of the *P.multocida* RPA-LFD assay, the same clinical sample (*n* = 95) set was evaluated by the qPCR assay, and result produced 0.958 kappa coefficient with qPCR (Table 4). However, the positive rate for isolation of *P. multocida* was lower than RPA-LFD assay in 95 clinical samples, [positive rate of 32.63% (31/95) and 54.74% (52/95), respectively]. The results showed that the culture method also had a lower clinical sensitivity than qPCR and RPA-LFD assays. In addition, previous antibiotic treatment of diseased animals may hinder bacterial isolation [13].

The RPA-LFD assay has the following merits: rapidity: RPA amplification and LFD detection can be finished within 30–40 min; isothermal amplification: operates at a lower temperature of 39 °C; and economical value: lower dependence on heat cycle instrument and professional operations. Combined with laboratory-independent nucleic acid purification technology, the RPA-LFD assay may be used as an alternative on-site diagnostic test in low-resource conditions [14, 15], since a simple heating system, such as water bath, chemical heater or even body heat could be used for the majority of the reaction [14–16]. Admittedly, the development of simplified DNA template preparation (NaOH-based DNA extraction) would further increase the potential for the wide utilization of this method [17]. Partial simulations of field conditions have provided evidence for the coming potential of the RPA-LFD assay as a sensitive, quick, and accurate field test for detection of *P. multocida*.

## Conclusions

In summary, the RPA-LFD was successfully applied for the detection of *P. multocida.* For the amplification reaction, only a water bath at 39 °C was needed, and amplicons analysis was simply carried out on the LFD. We have provided an evidence base for RPA-LFD as a tool for the sensitive, simple, and rapid detection of DNA from *P. multocida*, which potentially could be applied in clinical diagnosis and the molecular epidemiologic investigation of *P. multocida* infection.

## Methods

### Bacterial strains

*P. multocida* reference strains from serogroup A and B (Table 2) used in this study were provided from the BeNa Culture Collection (BNCC, China). Three capsular serogroups (D, E, and F) of *P. multocida* reference strains were provided from China Veterinary Culture Collection Center (CVCC, China). A total of 4 isolates of *P. multocida* previously isolated from cattle and goat and maintained at the Ruminant Disease Research Center, Shandong Normal University, Shandong Province, China, were used in this study. For the purpose of testing the detection specificity, members of the family Pasteurellaceae and other important bacterial pathogens of cattle which cause similar clinical signs (Table 2) were preserved in our lab or purchased from BNCC and employed.

### Clinical samples

A total of 95 clinical samples, including nasal swabs (*n* = 62) and fresh lung tissue of calves (*n* = 33), were collected from cattle with BRD on 17 different dairy farms located in 17 distinct geographic regions of Shandong province [18, 19] in China between January 2017 and February 2018 [20]. Samples were plated onto blood agar (5% fresh sheep blood) with a disposable sterile loop and incubated at 37 °C in air for a minimum of 48 h. All presumptive colonies of cultured *P. multocida* were further confirmed by PCR assay with primers specifically designed for the amplification of the *Kmt1* gene [9, 21]. DNA was extracted from the colonies and clinical samples by using the TIANamp bacteria DNA kit or swab DNA kit (Tiangen Biotech, China) according to the manufacturer’s instructions [5]. DNA was stored at − 20 °C until *P. multocida* detection could be performed.

### Design of RPA primer and LF-probe

**Fig. 3:**
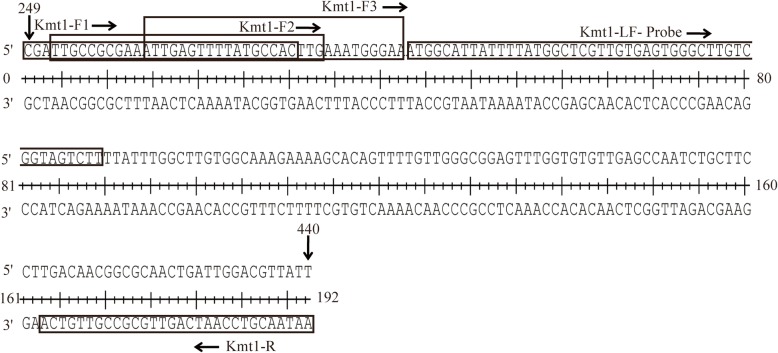
*P. multocida* RPA-LFD primer and LF-probe sequence. Location of the nucleotides was based on the sequence of the *Kmt1* gene

Three combinations of candidate primers (3 forward and one reverse) and one TwistAmp LF-probe (a 3′ blocker and an internal abasic site that replaces a nucleotide) were designed based on the sequence of *Kmt1* gene of *P. multocida* (nucleotides 249 to 440 of the GenBank accession number: AF016259.1) (Fig. 3 and Table 5). Synthesis was performed by TsingKe Biological Technology (Beijing, China).

**Table 5:** RPA primers and LF-probe designed in this study

Name	Sequence(5′-3′)	Genome location	Amplification size(bp)
Kmt1-F1	CGATTGCCGCGAAATTGAGTTTTATGCCAC	249–278	192
Kmt1-F2	TTGCCGCGAAATTGAGTTTTATGCCACTTG	252–281	189
Kmt1-F3	ATTGAGTTTTATGCCACTTGAAATGGGAA	262–290	179
Kmt1-R	Biotin-AATAACGTCCAATCAGTTGCGCCGTTGTCA	411–440	
Kmt1-LF Probe	FITC-ATGGCATTATTTTATGGCTCGTTGTGAGTG [dSpacer]GCTTGTCGGTAGTCTT-C3 Spacers	291–337	150
Pm-F1	CGATTGCCGCGAAATTGAGT	249–268	241
Pm-F2	CAGAGTTTGGTGTGTTGA	378–395	113
Pm-R	CAGACTGACAAGGAAATATAAAC	468–490	

*Abbreviations*: dSpacer is an exonuclease site, and C3 Spacers is a polymerase extension blocking site

### RPA-nfo reaction and optimisation

RPA-nfo reaction was carried out in a 50 μl volume using a ready-made TwistAmp nfo kit (TwistDX, UK) [5, 22]. For each sample, the rehydration solution was prepared as follows: 29.5 μl of 1 × rehydration buffer, 0.7 μl of FITC-labelled LF-probe, 2.0 μl of forward primer (10 μM), 2.0 μl of biotin-labelled reverse primer (10 μM), 10.9 μl of nuclease-free water, and 2.0 μl of *P. multocida* standard plasmid DNA or total DNA extracted from clinical samples. Then, 47.5 μl of the rehydration solution was transfered to the freeze dried reaction pellet and mixed by vortexing or pipetting up and down until the entire pellet had been resuspended. Finally, the reaction was initiated by adding 2.5 μl of magnesium acetate. The reaction tube was inserted into a thermostatic water bath and incubated at 38 °C for 30 min. The detection specificities of the different primer and LF-probe combinations were analysed. The RPA amplification products were purified using a universal UNIQ-10 column DNA purification kit (Sangon Biotech, China) then analysed by 2% AGE.

To define the optimum RPA reaction temperature, incubation temperatures ranging from 15 °C to 60 °C were tested in a thermostatic water bath. Then, incubation times ranging from 1 min to 40 min were assessed at the optimal reaction temperature.

### Lateral flow dipstick (LFD) assay

RPA amplification products were assessed by visualization using commercially available LFD (Milenia Genline Hybridetect-1 kits, Giessen, Germany). Dual-labelled RPA products had a 5′FITC label and a 5′biotin label on one strand and complementary strand attached to the anti-FITC gold [23]. The reaction products were visualized with dried gold particles conjugated to rabbit anti-FITC antibodies contained in the LFD at the application pad [24]; streptavidin (a biotin-ligand) was immobilized at the detection line. The DNA-gold conjugates were then captured at the biotin-ligand detection line. A control line functionalized with anti-rabbit antibody served as an assay control [23, 24]. Two microlitres of reaction solution were added to a new tube containing 98 μl PBST running buffer (PBS containing 0.1% Tween). Then, the sample pad end of LFD was vertically inserted into the buffer and maintained at room temperature for 5 min. After 5 min, the presence of the RPA product was shown by the development of a coloured test line.

### Analytical sensitivity and specificity of RPA-LFD

Next, positive standard plasmid DNA was constructed for use in RPA-LFD and qPCR (procedures consulting previous studies) using Pm-F1 and Pm-R primers (Table 5) [25]. The analytical sensitivity of both methods was compared using the standard plasmid DNA (10-fold serial diluted, 6 × 10^7^ to 6 copies/μl). A qPCR assay for *P. multocida* was carried out following previously reported procedures [26, 27]. The forward primer Pm-F2 and reverse primer Pm-R were used to amplify a 113-bp sequence between the 378–490 region of the *Kmt1* gene (Table 5). The analytical specificity of RPA-LFD was evaluated using DNA from 4 isolates of *P. multocida* and 14 reference strains of Pasteurellaceae in addition to other important bacterial pathogens of cattle which cause similar clinical signs (Table 2).

### Comparison of the RPA-LFD assay with the real-time qPCR or culture assay using clinical samples

The RPA-LFD assay for the detection of *P. multocida* was estimated by performing the assay on 95 clinical samples. The detection of clinical samples was performed in triplicate. The performance of the RPA-LFD assay was compared to that of qPCR and culture methods. The degree of agreement between the RPA-LFD and qPCR or culture assay results was measured according to the sensitivity, specificity, kappa value, positive predictive value (PPV) and negative predictive value (NPV) of each assay.

## Abbreviations

BRD: Bovine respiratory disease
Ct: Threshold cycle
HS: Haemorrhagic septicaemia
LFD: Lateral flow dipstick
NAAT: Nucleic acid amplification technologies
NPV: Negative predictive value
PCR: Polymerase chain reaction
PPV: Positive predictive value
qPCR: Real-time quantitative PCR
RPA: Recombinase polymerase amplification
